# Arthroscopic Biologic Tuberoplasty for Irreparable Rotator Cuff Tears: An Expedited Technique

**DOI:** 10.1016/j.eats.2022.08.035

**Published:** 2022-11-17

**Authors:** Misty Suri, Steven Parry, Manik Dham, Arjun Verma

**Affiliations:** a Ochsner Hospital for Orthopedics & Sports Medicine, New Orleans, Louisiana, U.S.A.; b Arthrex, Inc., Naples, Florida, U.S.A.

## Abstract

Massive irreparable rotator cuff tears in patients for whom arthroplasty is not an option can be a challenging clinical scenario for shoulder surgeons to manage. To achieve the best patient outcomes, a myriad of options has been presented in the literature, including debridement with biceps tenotomy or tenodesis, various tendon transfer procedures, superior capsular reconstruction, biceps tendon rerouting, bursal acromion resurfacing, balloon spacers, and tuberoplasty. While debridement with biceps tenotomy and superior capsular reconstruction have historically provided improvements in patient-reported outcomes, high rates of arthritis progression and failure of graft healing have been noted with these techniques, respectively. The superior capsular reconstruction has also proven to be technically challenging. The biologic tuberoplasty procedure was developed after several studies noted a lack of correlation between graft healing and improvement in patient-reported outcomes in superior capsular reconstructions, as long as the tuberosity remained covered with the graft. We present a technically efficient and expedited technique using an acellular human dermal allograft.

## Introduction

Massive irreparable rotator cuff tears present a challenging clinical scenario for shoulder surgeons, and there is significant controversy over which nonarthroplasty treatment options best optimize patient outcomes. Pain and dysfunction are thought to derive from pathoanatomic uncoupling of normal glenohumeral joint mechanics. This is generally observed with large rotator cuff tears, leading to proximal migration of humeral head and its bony articulation with the acromion during arm elevation and rotation.^1^ If left untreated, this can also result in wear of the glenohumeral cartilage, leading to rotator cuff tear arthropathy. Various options have been presented in the literature, including debridement with biceps tenotomy or tenodesis,^2^^,^^3^ various tendon transfer procedures,^4^^,^^5^ superior capsular reconstruction, biceps tendon rerouting,^6^ bursal acromion reconstruction,^7^ balloon spacers,^8^ and the tuberoplasty.^9^^,^^10^ The initial mini-open tuberoplasty procedure, as described by Fenlin et al., consists of rounding of the greater tuberosity to create a congruent acromiohumeral articulation, and it has been shown to have acceptable improvements in pain and function at a mean follow-up of 27 months.^11^ However, continued superior migration of the humeral head was noted, and further long-term studies are pending.^11^, ^12^, ^13^ Similarly, Scheibel et al. demonstrated that reversed arthroscopic subacromial decompression, including tuberoplasty, provided satisfactory to excellent results at a mean follow-up of 40 months.^14^ The procedure was subsequently modified by Mirzayan et al., who astutely noticed that in failed superior capsular reconstructions, clinical outcomes were still successful when the greater tuberosity remained covered by the dermal allograft.^15^ This finding led them to investigate a limited acellular dermal allograft resurfacing of just the greater tuberosity, thus providing an option for patients who are unwilling or unable to undergo shoulder replacement or more extensive arthroscopic operations due to medical comorbidities. The graft aims to serve as a bumper between the greater tuberosity and the acromion, thus preventing a painful articulation. We present our current technique for executing the biologic tuberoplasty procedure with modifications that may help to reduce surgical time and increase efficiency and reproducibility.

### Indications for Procedure

Biologic tuberoplasty is indicated in nonpseudoparalytic patients with preserved glenohumeral articular cartilage with massive irreparable rotator cuff tears. These patients have good passive range of motion with intact or repairable subscapularis and are typically poor candidates for arthroplasty due to young age or medical comorbidities. X-ray findings typical of an irreparable rotator cuff tear include superior migration of the humeral head or an acromiohumeral (AH) distance <7 mm.^16^ Irreparability is also suspected when preoperative magnetic resonance (MR) imaging demonstrates large multitendon tears with retraction to or past the glenoid with at least Goutallier Grade 3 atrophy.^17^, ^18^, ^19^ (Table 1) Advantages and disadvantages of the technique are listed in Table 2.

**Table 1:** Goutallier Classification of Fatty Degeneration in Rotator Cuff Tears

Grade 0 Completely normal muscle, without any fatty streaks
Grade 1 Some fatty streaks
Grade 2 Fat is present, but less fat than muscle
Grade 3 As much fat as muscle
Grade 4 More fat is present than muscle

**Table 2:** Advantages and Disadvantages

Advantages
1. Avoids risks of prosthetic replacement in poor candidates
2. Decreased time under anesthesia
3. Less technically challenging to reproduce than superior capsular reconstruction or bursal acromion resurfacing
4. Expedited rehab process
5. Pain relief
Disadvantages
1. Does not restore native shoulder biomechanics
2. Increased cost compared to debridement and biceps tenotomy/tenodesis or conventional tuberoplasty alone
3. It is possible the graft does not heal or becomes abraded by the acromion during arm movement

## Surgical Technique

The patient is placed in a lateral position using a bean bag with all bony prominences well padded. The arm is placed in a sterile arm positioner, which allows the surgeon to manipulate the arm intraoperatively. Longitudinal traction is applied to the arm to allow for opening of the subacromial space and glenohumeral joint. The arm is sterilely prepped and draped in the usual fashion.

A diagnostic arthroscopy is performed using standard posterior and anterior portals. The lateral portal is established, and the subacromial space is then entered. A subacromial decompression is performed as needed, with care taken to preserve the coracoacromial (CA) ligament. The rotator cuff is examined arthroscopically and manipulated to ensure that it is irreparable and that partial repair may be performed. Concomitant procedures, such as biceps tenodesis and distal clavicle excision, are also performed as deemed appropriate.

**Fig 1:**
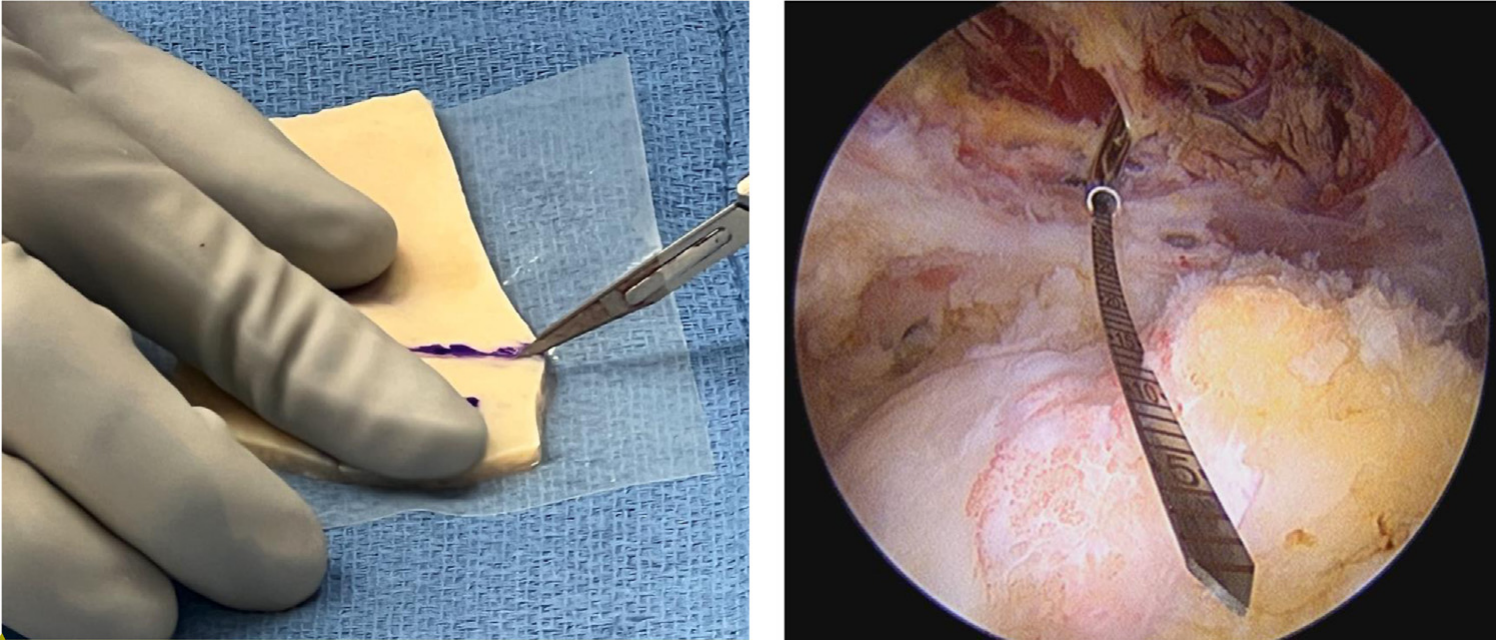
Graft site measurement (anterior to posterior). To measure the graft site, use a probe or SCR measuring guide.

**Fig 2:**
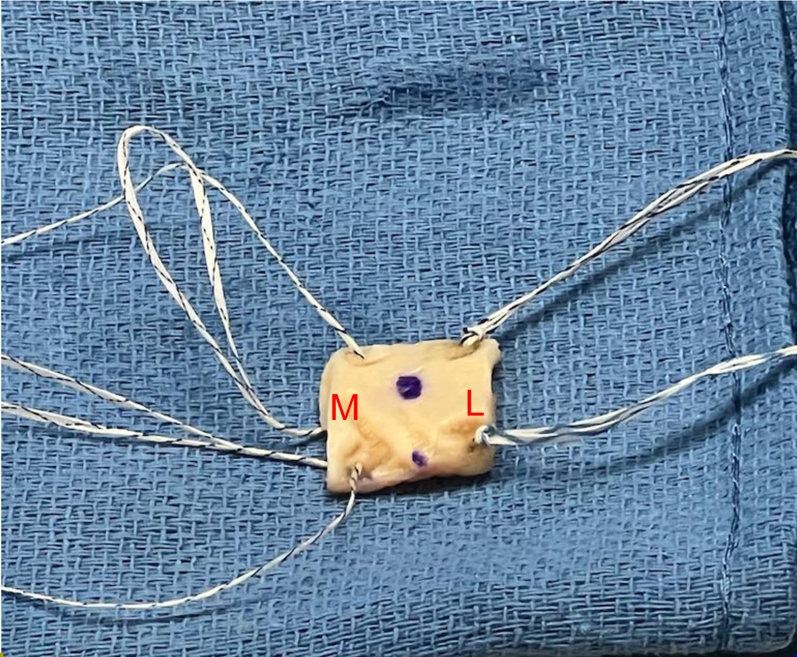
Final suture preparation of the graft. Medially, use suture passer to pass 0.9-mm suture tape. FiberLink sutures in the anterior and posterior medial corners. The loops should be placed on the. undersurface of the graft. Bring the loop closer to the graft (as this will be later used to shuttle. the repair suture from knotless FiberTak through the graft). Laterally, use suture passer to pass 1.0 × 1.3 SutureTape FiberLink and 1.0 × 1.3 SutureTape TigerLink in opposite corners of the graft laterally in a luggage tag configuration. The blue ink is shown on the superior side of graft. M, medial; L, lateral.

Following this, the area to be covered by the graft is measured in anterior-posterior and medial-lateral directions using an arthroscopic ruler. We prefer for the area of coverage to extend from the articular margin laterally past the edge of the greater tuberosity. The 3-mm thick acellular dermal allograft ArthroFLEX (LifeNet Health, Virginia Beach, VA) is then prepped by an assistant on the back table. The ArthroFLEX has two physically distinct sides: reticular and papillary; the rougher reticular side with its larger pores is to be the inferior surgical side. Note, the ArthroFLEX is packaged with papillary side visible through clear side of packaging. To ensure maintenance of orientation of the graft, mark the papillary (smoother and superior) side with sterile marker immediately after opening inner pouch. In preparation of the graft, we arthroscopically measure anterior to posterior (AP) defect and undersize the graft by 3-5 mm (15-20%) allowing for slight stretching of the graft (Fig 1A). Note, medial-lateral coverage is a relatively constant 20 mm. A FiberLink suture (Arthrex, Naples, FL) is then placed in the anteromedial and posteromedial corners of the graft, using a free needle, which will be used to shuttle the repair stitches from the medial row anchors through the graft. The anterolateral and posterolateral corners are prepped with 1.3 mm FiberLink SutureTape (Arthrex, Naples FL) in a luggage tag configuration (Fig 2).

**Fig 3:**
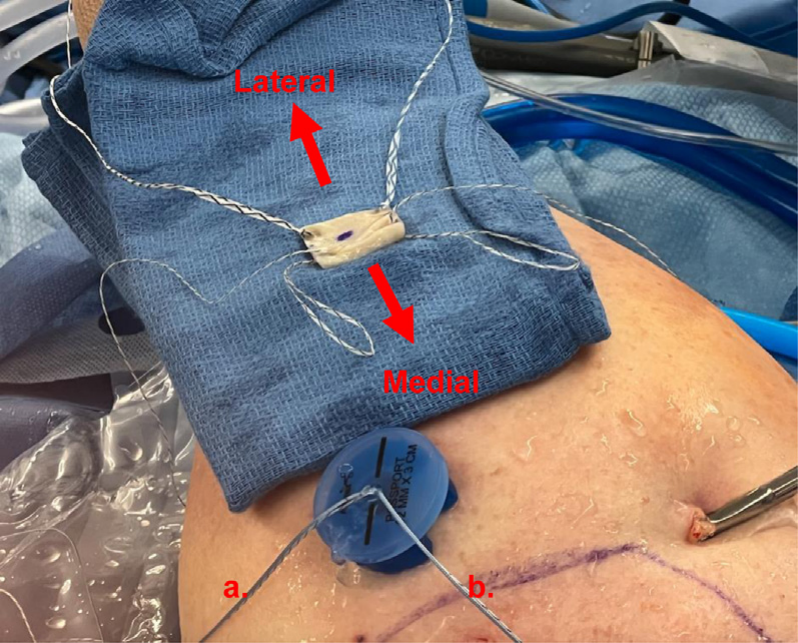
Arthroflex graft presented at the lateral side on the lateral shoulder. Note the anteromedial. and posteromedial 2.6 self-punching knotless FiberTaks have been inserted already at the corners. of the graft site. Next, through the lateral portal (12 mm × 3 cm passport), use a crab-claw grasper. to grasp the repair (blue) and the round shuttle loop from the anteromedial FiberTak anchor out. of the lateral portal. Clip these 2 limbs on the anteromedial (a) side of the passport. Repeat with. posteromedial anchor and clip the 2 suture limbs on the posteromedial (b) side of the passport cannula. Once the anchors have been inserted, keep the sutures on the relevant sides to prevent suture entanglement. Orientation: Right shoulder lateral position; anterior is left of figure, posterior is right of figure, proximal is bottom of figure, distal is top of figure. Lateral portal is the working portal with the passport cannula (12 mm × 3 cm) in place; and posterior portal is the viewing portal.

**Fig 4:**
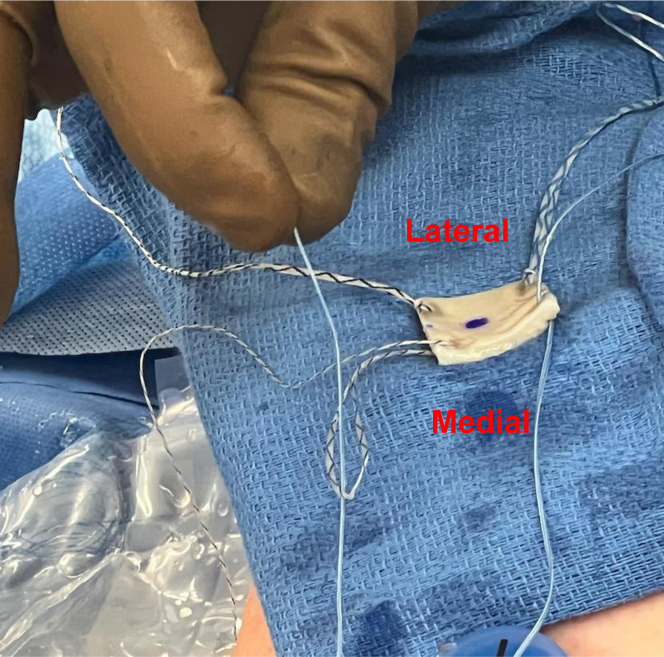
Bring the sutured graft on a sterile towel closer to the passport cannula with correct. orientation (medial side facing patients head). Use the anteromedial and posteromedial FiberLinks. to individually shuttle the repair suture (blue) from the knotless FiberTak anchors through the. Arthroflex graft. Note, the knotless mechanism is not yet “converted”.

**Fig 5:**
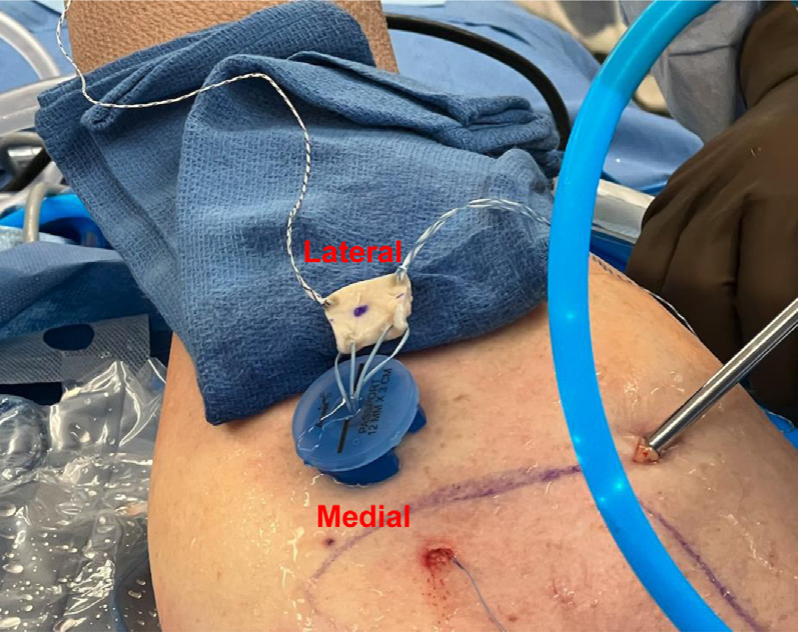
Once the repair sutures are passed through the graft, use the shuttle loop (on the same. sides) to “convert” the knotless mechanism. All repair stitches (blue) have been converted. through their respective knotless tensionable mechanism (anterior to anterior and posterior to. posterior). Now, the graft can be shuttled through the 12 mm × 3 cm passport cannula.

**Fig 6:**
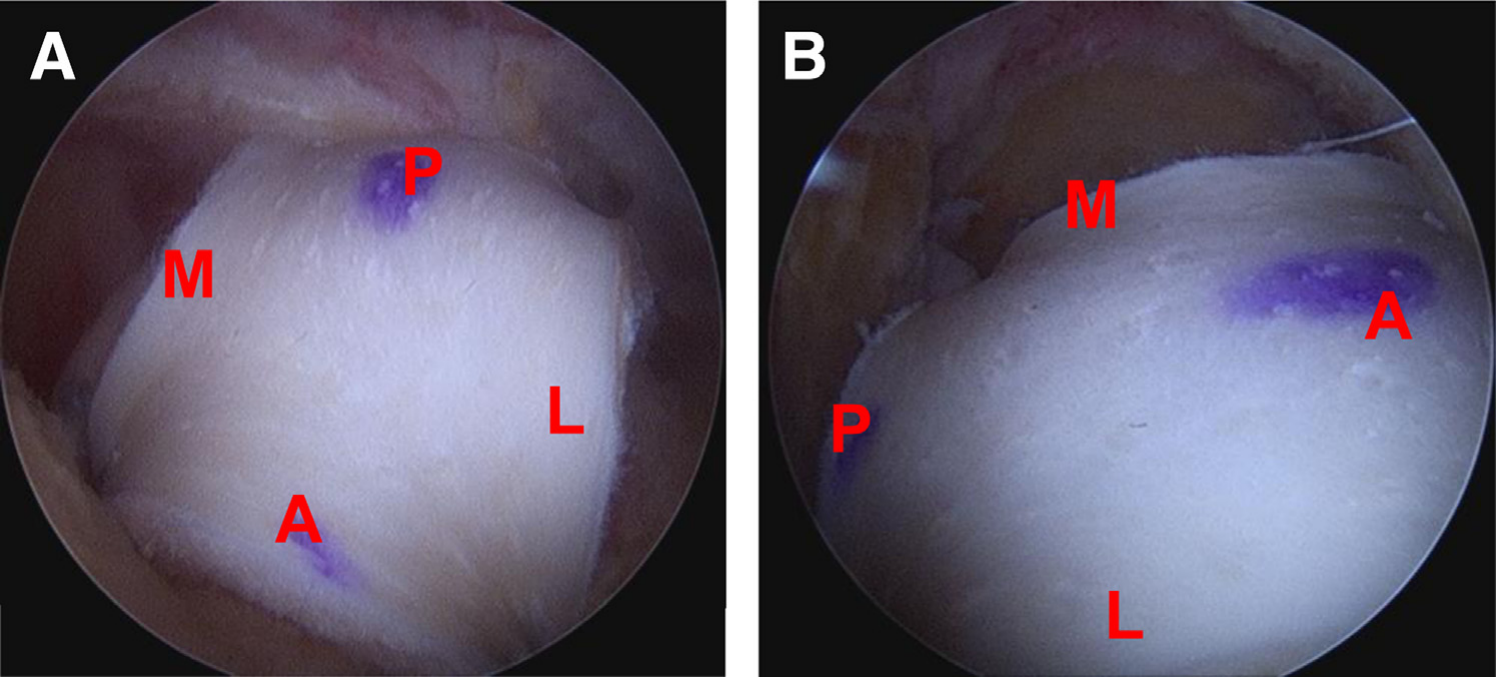
Posterior (A) and lateral (B) views of completed arthroscopic biologic tuberoplasty. M, medial; L, lateral; A, anterior; P, posterior.

Following this, an arthroscopic burr is used to prepare a bleeding bone bed with gentle decortication on the greater tuberosity. Anterior and posterior 2.6 self-punching knotless FiberTak anchors (Arthrex, Naples FL) are percutaneously, through separate portals, placed medially just off the articular margin (Video 1). The repair stitch and the looped shuttling stitch from each anchor are brought out through the lateral portal and clamped on its respective side of the lateral cannula to prevent suture entanglements (Fig 3). Anterior and posterior repair stitches are then passed through the graft using the previously placed FiberLink sutures (Fig 4). The repair stitches are then shuttled through the medial anchors using the looped stitch of the knotless tensionable mechanism (“converting” the repair stitch through the knotless mechanism) (Fig 5). The repair stitches are used to pull/shuttle the graft into place adjacent to the articular margin (Video 1). We prefer to fold the graft in a “taco” configuration and pass it through a 12-mm lateral passport cannula (Arthrex, Naples, FL), while providing gentle counter tension with the lateral luggage tagged sutures. Once the graft has been shuttled into place with a “ratcheting-type” alternating tensioning of the anterior and posterior repair stitches, the lateral repair stitches are loaded onto anterolateral and posterolateral 4.75-mm SwiveLock anchor (Arthrex, Naples FL). Free suture limbs from the medial anchors can be used to further re-tension if needed and then incised, leaving 2 mm of suture (Fig 6, Video 1). After the graft is secured into place, final confirmation of appropriate tensioning and graft position is confirmed from both posterior and lateral viewing portals (Fig 5). A complete list of pearls and pitfalls is listed in Table 3. Each of the crucial pieces of equipment used in this technique is listed in Table 4.

**Table 3:** Pearls and Pitfalls

Pearls
1. Very gentle decortication with the burr should be done. Any overaggressive decortication of the tuberosity should be avoided.
2. Undersize the graft by 2 to 3 mm (15-20%) in the anterior/posterior direction to allow for tensioning of the graft and compression to the tuberosity
3. Fold the graft in a “taco” configuration to pass through the lateral passport cannula.
4. Sequential tension the medial row knotless anchors as the graft is passed into the shoulder in a “ratcheting” or “back and forth”-type action to minimize chances of suture entanglement.
5. Keep gentle tension on the lateral row sutures as the medial sutures are tensioned to ensure smooth graft passage.
6. Ensure that all sutures are passed through the graft 3 mm or more from the edges to prevent suture pull out.
7. When tensioning the lateral row sutures, position the camera, so both the lateral anchor and the graft can be seen to ensure appropriate final tension as the anchor is set.
8. For the medial row, pass one repair stitch through the graft and then pass through the knotless mechanism before passing the other stitch through the graft (and then its respective knotless mechanism) to minimize chances of suture entanglement.
Pitfalls
1. Incorrect measurement of tuberosity or cutting the graft too small or too big
2. Overly aggressive decortication of the tuberosity may lead to anchor pullout.

**Table 4:** Crucial Pieces of Equipment

1. ArthroFLEX graft - AFLEX 301 (40 mm × 70 mm × 3 mm) 2. Sutures a. Lateral – 1 × 1.3 mm SutureTape FiberLink (AR-7535) i. 1 × 1.3 mm SutureTape Tigerlink (AR-7535T) b. Medial – 2 × # Fiberlink sutures (AR-7235) 3. Anchor Fixation a. Medial – 2 × Self Punching Knotless FiberTak anchors (AR-3641) b. Lateral – 2 × 4.75 Knotless SwiveLock Anchors 4. 1 × Passport Cannula 12 mm × 3 mm (AR-6592-12-30)

### Postoperative Protocol

The patient is placed in an abduction sling for 6 weeks postoperatively. Physical therapy starts at 7-10 days postoperatively and is initially geared toward pain reduction and passive range of motion with pendulum, Codman’s exercises, and periscapular function and activation. We allow weight bearing on the operative arm, as tolerated, and then allow active-assisted/active range of motion, as tolerated with strengthening to begin at 6 weeks postoperatively with a goal of pain-free range of motion by 3 months.

## Discussion

The concept of a biologic tuberoplasty stemmed from previous research on the superior capsular reconstruction (SCR), which has proven to be technically difficult to reproduce. Several recent articles have demonstrated radiographic graft healing in 38-81% of cases, with the majority of failures occurring on the glenoid side.^15^^,^^19^, ^20^, ^21^, ^22^, ^23^ However, in each of these investigations, authors noted that radiographic failure neither predicted nor correlated with worse outcomes compared to their healed counterparts. Ultimately, this led Mirzayan to develop the original biologic tuberoplasty technique, which entails a transosseous equivalent rotator cuff repair technique with three medial and three lateral row anchors with a crossing suture tape configuration.^10^ While effective, this technique does require a significant amount of suture management and, ultimately, operating room time.

Tendon transfers have also been proposed as options for irreparable rotator cuff tears, based on tear location and functional deficits.^5^^,^^24^ However, these have proven to be technically demanding, and outcomes in the literature have varied widely between authors.^25^

Another recent technique involves insertion of a saline-filled balloon into the subacromial space to act as a bumper and a humeral head depressor. Despite several small case series reporting benefit, Metcalf et al. recently published a double-blinded randomized controlled trial of 117 patients, which demonstrated greater improvements in the Oxford Shoulder Score in a debridement/biceps tenodesis group only vs. those who received an identical procedure with insertion of the subacromial balloon.^8^

The above technique, as described, aims to provide pain relief through arthroscopic placement of a biologic bumper between the greater tuberosity and the acromion, which can be performed expeditiously and efficiently in patients who are poor candidates for arthroplasty or more extensive arthroscopic procedures. In such cases, the bumper will help to maintain a degree of humeral head depression and aid in deltoid retraining by relieving pain. This technique is reproducible and efficient, and we believe it will result in similar rates of graft healing and pain control compared to Mirzayan’s technique.

Rehabilitation may be further expedited with this procedure, as compared to repair of a massive tear, tendon transfer, or superior capsular reconstruction. Although our preliminary results have been excellent, there is currently no long-term data on the procedure, and surgeons should proceed with this in mind.

## Supplementary Data

ICMJE author disclosure forms

Video 1 Arthroscopic biologic tuberoplasty for irreparable rotator cuff tears: an expedited technique.
